# A comprehensive dynamic growth and development model of *Hermetia illucens* larvae

**DOI:** 10.1371/journal.pone.0239084

**Published:** 2020-09-18

**Authors:** Murali Padmanabha, Alexander Kobelski, Arne-Jens Hempel, Stefan Streif

**Affiliations:** Automatic Control and System Dynamics Lab, Technische Universität Chemnitz, Chemnitz, Germany; Qinghai University, CHINA

## Abstract

Larvae of *Hermetia illucens*, also commonly known as black soldier fly (BSF) have gained significant importance in the feed industry, primarily used as feed for aquaculture and other livestock farming. Mathematical models such as the Von Bertalanffy growth model and dynamic energy budget models are available for modelling the growth of various organisms but have their demerits for their application to the growth and development of BSF. Also, such dynamic models were not yet applied to the growth of the BSF larvae despite models proven to be useful for automation of industrial production process (e.g. feeding, heating/cooling, ventilation, harvesting, etc.). This work primarily focuses on developing a model based on the principles of the afore mentioned models from literature that can provide accurate mathematical description of the dry mass changes throughout the life cycle and the transition of development phases of the larvae. To further improve the accuracy of these models, various factors affecting the growth and development such as temperature, feed quality, feeding rate, moisture content in feed, and airflow rate are developed and integrated into the dynamic growth model. An extensive set of data was aggregated from various literature and used for the model development, parameter estimation and validation. Models describing the environmental factors were individually validated based on the data sets collected. In addition, the dynamic growth model was also validated for dry mass evolution and development stage transition of larvae reared on different substrate feeding rates. The developed models with the estimated parameters performed well, highlighting their potential application in decision-support systems and automation for large scale production.

## Introduction

*Hermetia illucens*, commonly known as the black soldier fly (BSF), is an insect species which is widely studied for the high nutrition value of its larvae. Studies [1–4], showcase these nutritional values and its suitability as a source for animal feed and human food. Several studies, [5] and [6–9] amongst the recent, also indicate their application for recycling food and bio waste. These studies clearly demonstrate the potential of *Hermetia illucens* in addressing the approaching food scarcity while reducing the resource usage for their production. Irrespective of the potential applications of *Hermetia illucens*, for their (mass) production, it is necessary to study: (1) the underlying biological processes such as assimilation, respiration, morphological changes, etc.; (2) the fundamental resource prerequisites such as feed composition, growing environment conditions, etc.; (3) the resulting growth dynamics that exhibits the various stages of the larval growth in response to the supplied resources; and (4) the interaction between the larvae and its environment (microbiome, substrate, etc.) and biological effects that trigger certain events (e.g. fleeing from substrate due to low *O*_2_ concentration etc.).

This insect species originates from tropical South American climate zones and thus requires a warm and humid environment. Such conditions were verified in research studies: [10] highlighted the threshold temperatures and thermal requirements; [11] compared the development rates over different temperature ranges; and [12] studied the effect of humidity on the egg eclosion and adult emergence. The influence of diet, its moisture content and the temperatures were studied together to showcase its importance in the development of the larvae [13–16]. Another study [17], proposed and showed the effects of pH levels of the substrate (feed), in which larvae are grown, on the larval development. From these studies, one can conclude the importance of the environmental conditions (temperature, humidity, etc.), the substrate conditions (moisture, pH, etc.) and the feed composition for the growth and development of the larvae.

A thorough literature survey revealed only time-invariant static models that describe certain biological processes of the BSF larvae. The authors of [10] suggested a model to describe the development rate as a function of temperature and, similarly, a model for calculation of metabolic rate as a function of temperature was presented in [18]. In case of [19], a logistic model was suggested for modelling the larval growth in response to the air flow rate. Also, a more recent work [20] suggested the use of a Richards model to fit the larval growth. These models from literature are mostly static models and do not adequately describe various time-dependent dynamical aspects of larvae production such as resource dynamics, environment dynamics, etc. Also, it can be observed that the motivation behind the above mentioned literature was to improve the growth and hence the large scale production of BSF larvae. In order to fully utilize such models for performing simulation studies, reactor design, process design, automation, control and resource optimization, it is also necessary to appropriately formulate them as dynamic models. The main aim of this work is to develop suitable mathematical models that adequately describe the effect of environmental conditions on the larval metabolism; the larval growth describing the evolution of its dry mass over time; and finally, the transition of development stages between larvae and pupae.

The following sections provide in detail the approach taken to develop the models, analyze the data and obtain the model parameters. Firstly, a detailed explanation of the experimental setup is provided. Then, a dynamic model describing the growth and development of the larvae is presented. The experiments performed for the estimation of parameters are described followed by the model parameter estimation. Finally, the results of the models are compared with the actual measurement data and the quality of fit is determined for the models.

## Materials and methods

In this work, data for model development, parameter estimation and model validation are mainly obtained from literature and experiments performed in this study. Details of the experiments performed and the data source are also provided. The following sections provide the details of the mathematical models developed in this work and the procedure followed for estimating the model parameters.

### Production unit

The studies on the production of larvae in an artificial controlled environment in this work are conducted in a custom built production unit [21] that can provide the necessary growing conditions and simultaneously perform measurements of various parameters (e.g. air and substrate temperature, CO_2_ and O_2_ concentrations, humidity). The controlled environment has a volume of 75 L and holds a growing tray of dimension 22 cm × 32 cm × 5.5 cm that could contain up-to 4 kg of substrate. This growing tray serves as a container for the growing medium that contains selected feed for the larvae, selected number of young larvae (neonates) and the microbiome that eventually develops and grows along with the larvae in the growing medium. The temperature, humidity, airflow/air-concentration, and day-night cycles/photo period within the unit can be regulated as required. Information related to the states inside the production unit such as temperature of air and growing medium; CO_2_ and O_2_ concentrations; and humidity in air and moisture in growing medium are recorded by the sensors integrated within. Similarly, information related to the states outside the production unit, e.g., temperature, humidity and CO_2_ concentration of external air source, are logged using data loggers. Further details regarding the production environment can be found in [21] (see Section 2.1.4 and 3.7).

### Experiment setup for moisture dependency

To study the dependency on substrate or feed moisture, a larval growth experiment was performed. In this experiment, ten small containers of height 8 cm and diameter 5 cm were filled with 10 g of dry feed and varying amounts of water, from 0 to 40 g, were added. Then, 20 larvae of about 8 days old with a starting weight of 2 mg were added to each container. A small net was placed over each container to prevent the larvae from escaping while allowing air exchange. All containers were then placed inside the production unit with air temperature set at 29°C and air ventilation at a rate of 7.5 l min^-1^. The weight of each container was checked daily. Any changes in container weight, considered mostly due to evaporation, was supplemented to keep the moisture constant. The final fresh and dry weight of the larvae (dried for 6 h at 70°C in an air dryer) was measured at the end of the experiment (on 8th day).

### Modelling approach for larvae growth, development and the influence of its environment

The main focus of this work is to obtain a model that describes the evolution of dry mass over a given period in response to various environmental factors. These models should also capture the drop in larval dry mass due to the maturation process that BSF larvae undergo during their last larval instars. Furthermore, it is also necessary to obtain information related to the development phases of the larvae that can be used for streamlining the production process. This description can be assistive in determining harvesting strategies such as harvesting for maximum larval dry weight or for obtaining pupae for rearing adults.

The most commonly used models to describe the growth of biological organism, among others, are the von Bertalanffy growth model [22] and dynamic energy budget (DEB) [23] model. The former model describes the growth empirically while the latter is based on mechanistic description using the concepts of energy reserves and volume. Despite having a simple model structure, the von Bertalanffy model can be used to model the dry weight/size change over time. However, no inference can be obtained regarding the current development phase of the larvae or the drop in dry weight during maturation. The DEB model in comparison, uses states (energy density and structural volume) that are either difficult or not directly measurable. Also, it is not evident if using this model, information pertaining to the development phases could be obtained. Therefore, in this work, a new model is developed based on the mass balance approach and uses concepts such as asymptotic maximum size proposed for use with von Bertalanffy model [24] and the concept of maturity reserves used in DEB model. The following section provides some background to the fundamentals of larval growth and an overview of the model development based on these fundamental principles. Table 1 lists all the symbols used in this work for developing the models.

**Table 1:** List of symbols used in the description of the models.

Symbol	Description	Unit
*B*_dry_	dry mass per larva	[g]
*B*_wet_	wet mass per larva	[g]
*B*_eff_	non structural assimilates in larva	[g]
*B*_str_	structural mass of the larva body	[g]
*T*_Σ_	development sum of larvae from neonates to prepupa	[h]
*B*_feed_	total feed (dry mass) available in the growing medium	[g]
*T*_med_	temperature of growing medium in production unit	[°C]
*W*_med_	total water in the growing medium	[kg]
*W*_med%_	moisture concentration of substrate	[kg kg^-1^]
*C*_air_	CO_2_ concentration of air in production unit	[kg m^-3^]
*O*_air_	O_2_ concentration of air in production unit	[kg m^-3^]
*H*_air_	absolute humidity of the air in production unit	[kg m^-3^]
*A*_air_	air flow rate to the larvae production unit	[l min^-1^]
$\phi_{B_{\text{ing}}}$	flux of feed from substrate into the larva	[g s^-1^]
$\phi_{B_{\text{excr}}}$	flux of non digested feed back to substrate	[g s^-1^]
$\phi_{B_{\text{assim}}}$	feed converted into energy and spent to digest the ingested feed	[g s^-1^]
$\phi_{B_{\text{mat}}}$	assimilates spent towards building of new structure	[g s^-1^]
$\phi_{B_{\text{maint}}}$	assimilates spent for maintenance of existing structure	[g s^-1^]
$\phi_{B_{\text{eff}}}$	effective assimilates available from the ingested feed for growth and maintenance	[g s^-1^]
$\phi_{B_{\text{metab}}}$	total assimilates spent for metabolic activities	[g s^-1^]
$k_{\alpha_{\text{excr}}}$	fraction of ingested feed excreted out	[-]
$k_{\alpha_{\text{assim}}}$	fraction of ingested spent for digestion	[-]
*ϵ*_inges_	efficiency of the ingested feed	[-]
*k*_inges_	specific ingestion rate of larva	[g g^-1^ s^-1^]
*k*_maint_	specific rate of maintenance and maturity of larva	[g g^-1^ s^-1^]
$k_{{\text{dev}}_{\text{ts}}}$	conversion factor to obtain development sum in hours	[s^-1^]
*k*_T_Σ_1_	development point at which the assimilation process starts to cease	[h]
*k*_T_Σ_2_	development point at which the assimilation process ends	[h]
*k*_T_Σ_3_	development point at which the larval development phase ends	[h]
$k_{B_{\text{asy}}}$	asymptotic size of the larvae in dry mass	[g]
$k_{T_{L}}$	lower boundary temperature for Arrhenius equation	[K]
$k_{T_{\text{ref}}}$	reference temperature for Arrhenius equation	[K]
$k_{T_{H}}$	upper boundary temperature for Arrhenius equation	[K]
$k_{T_{\text{AL}}}$	Arrhenius temperature for the lower boundary temperature $k_{T_{L}}$	[K]
$k_{T_{A}}$	Arrhenius temperature for the reference temperature $k_{T_{\text{ref}}}$	[K]
$k_{T_{\text{AH}}}$	Arrhenius temperature for the upper boundary temperature $k_{T_{H}}$	[K]
*k*_r_ref_T_	development rate observed at the known reference temperature $k_{T_{\text{ref}}}$	[s^-1^]
*k*_r_max_T_	maximum observed development rate in response to temperature (Logan-10 model)	[s^-1^]
*k*_r_base_T_	minimum development rate observed above the lower temperature boundary (Logan-10 model)	[s^-1^]
*k*_*ρ*T_	development rate change per degree change in temperature (Logan-10 model)	[°C^-1^]
$k_{T_{\text{base}}}$	lower temperature boundary above which the development is observed (Logan-10 model)	[°C]
$k_{T_{\text{max}}}$	lethal maximum temperature for larval survival (modified Logan-10 model)	[°C]
*k*_ΔT_	width of the high temperature boundary (modified Logan-10 model)	[°C]
*k*_r_max_dm_	maximum development rate of the larvae in response to feed density/availability	[s^-1^]
*k*_B_half_dm_	feed density/feeding rate fow which the development rate is half	[g g^-1^ d^-1^]
*k*_r_max_gm_	maximum growth rate of the larvae in response to feed density/availability	[g s^-1^]
*k*_B_half_gm_	feed density/feeding rate for which the development rate is half	[g g^-1^ d^-1^]
*k*_r_max_w_	maximum growth rate in response to feed moisture concentration	[g d^-1^]
*k*_W_med_C1_	lowest feed moisture below which the growth ceases	[g g^-1^]
*k*_W_med_C2_	feed moisture above which the ingestion rate can reach maximum	[g g^-1^]
*k*_W_med_C3_	feed moisture above which the diffusion of oxygen/air exchange starts to cease	[g g^-1^]
*k*_W_med_crit_	feed moisture above which the larvae begins to die	[g g^-1^]
*k*_A_inf_A_	infliction point for logistic model at which the growth rate is half for given airflow rate	[l min^-1^ g^-1^]
*k*_A_trans_A_	airflow rate influenced growth rate transition range for logistic model	[l min^-1^ g^-1^]
*k*_r_max_A_	maximum observed growth rate in response to airflow rate	[g s^-1^]
$k_{A_{\text{half}}}$	air flow rate for which the growth rate is reduced to half	[l min^-1^]
*r*_assim_	regulation of assimilation rate in response to internal and external factors	[-]
*r*_mat_	regulation of maturity-maintenance rate in response to internal and external factors	[-]
*r*_dev_	regulation of development rate in response to external factors	[-]
*r*_T_	larva development rate in response to substrate temperature	[s^-1^]
*r*_F_	larva development rate in response to feed density	[s^-1^]
$r_{F_{\text{grw}}}$	larva growth rate in response to feed density	[g s^-1^]
*r*_W_	larva growth rate in response to substrate moisture	[g s^-1^]
$r_{W_{\text{assim}}}$	larva assimilation rate change in response to substrate moisture	[-]
$r_{W_{\text{resp}}}$	larva respiration rate change in response to substrate moisture	[-]
*r*_A_	larva growth rate in response to air flow rate	[g s^-1^]
$r_{B_{\text{assim}}}$	change of assimilation rate in larva over its development period	[-]
$r_{{\text{assim}}_{\text{max}}}$	change of ingestion potential of larva with its dry mass	[g g^-1^]
$r_{B_{\text{mat}}}$	change of maturity-maintenance rate in larva over development period	[-]

#### Larvae growth and dry mass partitioning

The collective processes that define the growth and development of an organism are known to be the metabolism. The important processes of the metabolism in an organism—here abstracted—are assimilation, maintenance, growth, maturity. Using the mass/energy balance approach, these abstracted metabolic processes can be used to describe the growth and development rate of an organism. General consideration in this approach is that the afore mentioned abstract processes can either use energy or mass for developing the model. Since mass is easy to measure in-situ both in laboratory and in production compared to measuring energy, in this work models are derived based on the mass.

**Fig 1:**
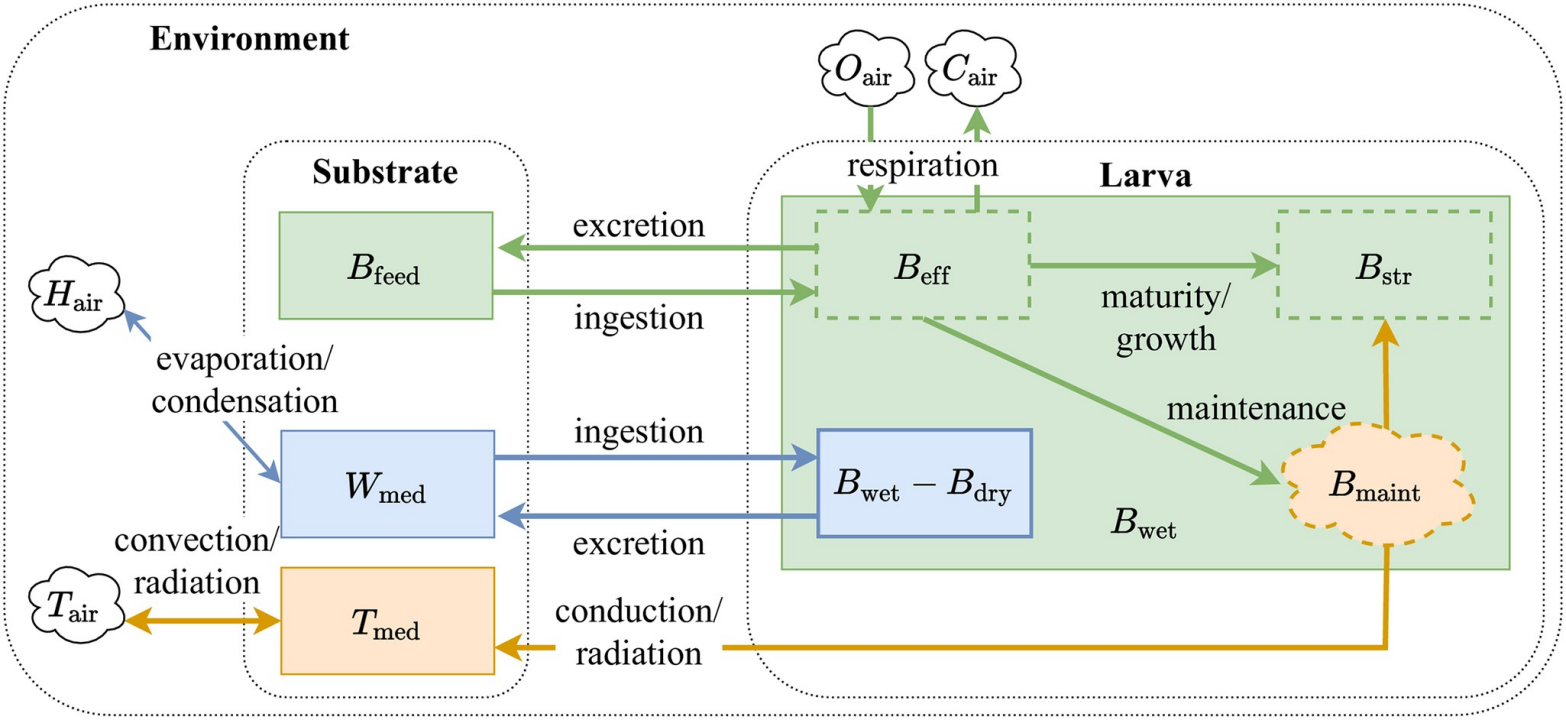
Mass and energy flow between the larva, substrate and the growing environment. The rectangles represent the different states and the arrows indicate the flow of mass and energy (fluxes) between these states. Biomass and water in the substrate enters and exits larvae by ingestion and excretion. Gas exchange as a result of metabolic respiration takes place between the larva and the environment. Assimilated biomass and reserves *B*_eff_ is further converted into structure towards the larval maturity *B*_str_ and energy *B*_maint_ necessary for maintenance of the structure. The states represented in dashed lines indicate that they are not directly measurable unlike the larva wet and dry mass *B*_wet_ and *B*_dry_ respectively. A part of the *B*_maint_ is converted to heat, a byproduct of metabolism, and is lost to the substrate increasing its temperature *T*_med_.

Growth, which describes the increase in structural volume or mass in an organism, requires ingestion of feed. This feed ingested by larva is converted into assimilates through the process of assimilation consuming a portion of the assimilates for this process. The assimilates are converted into structural mass towards growth and maturity. Maturity, which is an indicator for larval development, consumes assimilates throughout the larval stage. Maintenance respiration that keeps the organism alive also consumes some of the assimilates. Using a Forrester diagram, the flow and partitioning of biomass and energy through the afore mentioned processes are presented in Fig 1.

With this context and background, growth or the rate change of the larval dry mass is represented using mass balance model as

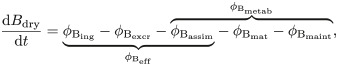
(1)
where $\phi_{B_{\text{ing}}}$ is feed flux from substrate into the larvae, $\phi_{B_{\text{excr}}}$ is the flux of non digested feed back to the substrate, $\phi_{B_{\text{assim}}}$ is the feed converted into energy necessary for assimilation of the ingested feed, $\phi_{B_{\text{maint}}}$ is the assimilates converted into energy for basal maintenance of existing structure, $\phi_{B_{\text{mat}}}$ is the assimilates spent for growth and maturity (responsible for accumulating new structure) and $\phi_{B_{\text{metab}}}$ represents all the assimilates consumed for metabolic activities.

The effective assimilates available for growth and maintenance $\phi_{B_{\text{eff}}}$ can be expressed as

$$\begin{array}{c} \phi_{B_{\text{eff}}}=\underset{\epsilon_{\text{inges}}}{\underset{︸}{\left(1-k_{\alpha_{\text{excr}}}-k_{\alpha_{\text{assim}}}\right)}}\;\phi_{B_{\text{ing}}}, \end{array}$$ (2)

where $k_{\alpha_{\text{excr}}}$ and $k_{\alpha_{\text{assim}}}$ are respectively the fractions of feed excreted and spent in the process respectively and *ϵ*_inges_ corresponds to the efficiency of the digested feed and provides information related to the quality of the feed.

Furthermore, maturity and maintenance expenses ($\phi_{B_{\text{mat}}}$ and $\phi_{B_{\text{maint}}}$) cannot be distinguished since these two processes are active during the entire development phase of the larvae [16]. Due to this reason as well as the terms being difficult to separately measure, they are combined into one flux term. These flux components corresponding to the assimilation and maintenance are considered proportional to the weight/size of the organism [22]. Therefore substituting Eq (2) in Eq (1), replacing $\phi_{B_{\text{ing}}}$ with *k*_inges_ *B*_dry_, and replacing $\phi_{B_{\text{mat}}}+\phi_{B_{\text{maint}}}$ with *k*_maint_ *B*_dry_, Eq (1) can be rewritten in terms of dry weight as

$$\begin{array}{c} \frac{dB_{\text{dry}}}{dt}=\epsilon_{\text{inges}}\;k_{\text{inges}}\;B_{\text{dry}}-\;k_{\text{maint}}\;B_{\text{dry}}, \end{array}$$ (3)

where *k*_inges_ and *k*_maint_ are the specific maximum ingestion rate and specific maximum maturity and maintenance rate respectively (g feed g^−1^ larvae s^−1^ in dry matter). The Eq (3) is of the form similar to the general form of von Bertalanffy model given in Eq (5) of [22] with *m* = 1 for insects as suggested in [25]. This model given by Eq (3) is rudimentary, describing only partitioning of the biomass across different biological processes. To achieve the model goals described previously, it is also necessary to model different factors such as temperature, feed quality, current larval instar, etc., that regulate the rate of flow of the mass and energy fluxes across different processes. Introducing the factors influencing the growth, Eq (3) can be reformulated as

$$\begin{array}{c} \frac{dB_{\text{dry}}}{dt}=\epsilon_{\text{inges}}\;r_{\text{assim}}\;k_{\text{inges}}\;B_{\text{dry}}-\;r_{\text{mat}}\;k_{\text{maint}}\;B_{\text{dry}}, \end{array}$$ (4)

where the functions *r*_assim_ and *r*_mat_ regulate the rate of assimilation and maturity-maintenance respectively in response to the current development stage and the available growing conditions. Deriving these regulation functions and identification of the factors affecting the growth and development is necessary. However, it is first necessary to identify a mechanism to model the development process using which the development phases of the larvae can be tracked.

#### Larvae development

Growth process in larvae takes place in stages which are commonly known as instars with the *Hermetia illucens* larvae undergoing a total of 6-7 instars [26, 27]. This development, however, seems to be not dependent on the size of the larvae. This can be inferred from the data presented in [13], where the larvae completed their development despite not reaching nearly half their maximum size. Therefore, an alternate mechanism is required to model the developmental stages of the larvae. Using this mechanism the last two larval instar stages, which have significant influence on the growth process, can be tracked.

The use of temperature sums or degree days to track the developmental stages and growth of an organism, including plants and insects, can be seen in literature [28–30]. For organisms growing in open fields where mostly only temperature vary, it was possible to estimate the actual development stage by tracking the total suitable heat the organism received during its lifetime. This concept of temperature sums serves as a unit to calculate the apparent age of the organism [31] that is different from the real age which is the time since the larva is hatched. However, this might only work for cases where the resources such as food, air concentration and heat are not limited. Therefore, as also suggested in [31], not only temperature but also other environmental conditions such as feed density, air concentration etc., need to be considered to obtain the apparent age that serves as an indicator to the total energy received by the larvae in its lifetime. In this work, such indicator for total energy is obtained from the total number of hours that the larva receives suitable growing conditions (apparent age) in its lifetime (real age).

Similar to integrating the temperatures over time as in degree days, here the instantaneous development rates determined by the environmental conditions are integrated. This integrated sum of development rates—referred to as development sum *T*_Σ_—is introduced. This development sum can therefore be written as a function of all factors that affect the development rate as

$$\begin{array}{c} \frac{dT_{\Sigma }}{dt}=r_{\text{dev}}\left(T_{\text{med}},B_{\text{feed}},W_{\text{med}},A_{\text{air}}\right)\;k_{{\text{dev}}_{\text{ts}}} \end{array}$$ (5)

where *r*_dev_ is the function regulating the development rate for the available given growing conditions such as temperature in substrate *T*_med_, feed availability *B*_feed_, moisture in substrate *W*_med_, air flow rate *A*_air_ and $k_{{\text{dev}}_{\text{ts}}}$ is the maximum rate at which the apparent age (in h) changes in relation to the real age. This Eq (5) now provides a new unit of measure for apparent age to identify the current development stage.

#### Effect of external factors on larval growth and development

Factors that affect the growth and development of the larvae considered in this work include temperature, feed density, feed quality, moisture and air concentration. Each of these parameters influence the larval development through various biological processes. An attempt is made to model these influences through mechanistic and analytical models both from literature and developed based on the analysis of aggregated literature and experiment data. The factors affecting the growth and development are studied and modelled independently by varying only one factor and keeping the other factors constant.

*Temperature*. Temperature has a direct effect on all the biochemical reactions that take place in the larvae and thus affecting its growth and development rate. The effect of temperature on the growth of larvae can be modeled using Arrhenius equation [32]. Corrections to the metabolic rates for the temperatures beyond the upper and lower boundaries can be applied as

$$r_{\text{T}}(T_{\text{med}})=\frac{k_{r_{\text{ref}}\text{T}}\text{exp}(\frac{k_{{\text{T}}_{\text{A}}}}{k_{{\text{T}}_{\text{ref}}}}-\frac{k_{{\text{T}}_{\text{A}}}}{T_{\text{K}}})}{\left(1+\text{exp}(\frac{k_{{\text{T}}_{\text{AL}}}}{T_{\text{K}}}-\frac{k_{{\text{T}}_{\text{AL}}}}{T_{{\text{T}}_{\text{L}}}})+\text{exp}(\frac{k_{{\text{T}}_{\text{AH}}}}{T_{{\text{T}}_{\text{H}}}}-\frac{k_{{\text{T}}_{\text{AH}}}}{T_{\text{K}}})\right)}$$ (6)

with *T*_K_ = *T*_med_ in K, where $k_{T_{\text{ref}}}$ is the reference temperature for which the development rate is known, $k_{T_{A}}$, $k_{T_{\text{AL}}}$ and $k_{T_{\text{AH}}}$ are Arrhenius temperatures at reference $k_{T_{A}}$, lower boundary $k_{T_{L}}$, and upper boundary $k_{T_{H}}$ temperatures respectively, and *k*_r_ref_T_ is the known reference development rate.

Another analytical model proposed in [33] (see Eq (10) of [33]), referred to in this work as Logan-10, also describes the growth rate in response to the temperature as well considering the effects of denaturation and desiccation at high temperatures. The Logan-10 model was modified such that for temperatures beyond the upper threshold, the resulting growth is zero instead of a negative growth. The resulting modified Logan-10 model is given as

$$\begin{array}{c} r_{T}\left(T_{\text{med}}\right)=k_{r_{\text{max}}T}{\left(1+k_{\gamma }\exp(-k_{\rho }T(T_{\text{med}}-k_{T_{\text{base}}}))+\exp(-\frac{k_{T_{\text{max}}}-T_{\text{med}}}{k_{\Delta T}})\right)}^{-1}, \end{array}$$ (7)

with $k_{\gamma }=(\frac{k_{r_{\text{max}}T}-k_{r_{\text{base}}T}}{k_{r_{\text{base}}T}})$, where *k*_r_max_T_ is the maximum observed rate (s^-1^), *k*_r_base_T_ is the minimum rate at the temperature above the lower threshold, *k*_*ρ*T_ rate change in response to the temperature, $k_{T_{\text{max}}}$ is the lethal maximum temperature and *k*_ΔT_ is the width of the high temperature boundary layer. The Logan-10 model has one less parameter compared to Arrhenius model presented in Eq (6) and can be intuitively approximated from the available data. In this work, both these models will be evaluated and the corresponding parameters will be estimated.

*Feed density*. The feed flux assimilated by the larvae is effected by few factors, considered important in this work due to its application, such as feed availability and change in feeding behavior due to the modifications to the mouth parts of the larvae in its final instars. It is common in literature to model the change in ingestion rate due to substrate availability using a type II function. Monod presented an adaptation of this function to model the growth of bacterial cultures in [34]. This Monod equation is adapted in this work as

$$\begin{array}{c} r_{F}\left(B_{\text{feed}}\right)=k_{r_{\text{max}}\text{dm}}\frac{B_{\text{feed}}}{B_{\text{feed}}+k_{B_{\text{half}}\text{dm}}}, \end{array}$$ (8)

where *B*_feed_ is the feed density in the substrate/growing medium, *k*_r_max_dm_ is the maximum development rate (s^-1^) at highest feed density and *k*_B_half_dm_ is the feed density resulting in half of the maximum rate.

Similarly, Eq (8) can be rewritten to also model the growth rate of the larvae as

$$\begin{array}{c} r_{F_{\text{grw}}}\left(B_{\text{feed}}\right)=k_{r_{\text{max}}\text{gm}}\frac{B_{\text{feed}}}{B_{\text{feed}}+k_{B_{\text{half}}\text{gm}}}, \end{array}$$ (9)

where *k*_r_max_gm_ is the maximum growth rate (g s^-1^) of the larvae and *k*_B_half_gm_ is the feed density resulting in half of the maximum growth rate.

*Feed moisture*. Based on the data presented in [19], the authors suggest that the moisture of the feed (kg water in kg wet feed) has a first order effect. However, in that study, data was only available for the moisture content of 48-68%. Another early work studied the development of different flies, including *Hermetia illucens*, under different substrate moisture conditions [35]. In this work, the authors concluded that the development increased with increase in moisture content between 30-70%, while, at 20, 80 and 90% there was no development observed. The moisture experiment performed as part of this work, covered the feed moisture in the very low and high concentrations. Based on these results, the feed moisture has different influences on the larval growth. Firstly, with lower moisture, the feed may not be ingestible and thus result in slower growth and high mortality rate at very low moisture levels. Secondly, with increasing moisture, feed could be assimilated better resulting in better growth. Finally, at higher moisture concentrations, intake of oxygen might be reduced resulting in slower growth and higher larval mortality.

Based on these observations, the influence of water content in feed on the larval growth can be modelled as

$$r_{\text{W}}(W_{\text{med}\%})=k_{{\text{r}}_{\text{max}}\text{W}}\;r_{{\text{W}}_{\text{assim}}}(W_{\text{med}\%})\;r_{{\text{W}}_{\text{resp}}}(W_{\text{med}\%}),$$ (10)

where *k*_r_max_W_ is the maximum growth rate, and the influence of moisture content on the assimilation rate and respiration rate $r_{W_{\text{assim}}}$ and $r_{W_{\text{resp}}}$ respectively are modelled as

$$\begin{array}{cc} r_{W_{\text{assim}}}\left(W_{\text{med}\%}\right) & =\{\begin{array}{cc} \;0 & \text{if}\;W_{\text{med}\%}\leq k_{W_{\text{med}}C1}\\ \frac{W_{\text{med}\%}-k_{W_{\text{med}}C1}}{k_{W_{\text{med}}C2}-k_{W_{\text{med}}C1}} & \text{if}\;k_{W_{\text{med}}C1}<W_{\text{med}\%}<k_{W_{\text{med}}C2}\\ \;1 & \text{if}\;W_{\text{med}\%}\geq k_{W_{\text{med}}C2} \end{array}, \end{array}$$ (11)

$$\begin{array}{cc} r_{W_{\text{resp}}}\left(W_{\text{med}\%}\right) & =\{\begin{array}{cc} \;1 & \text{if}\;W_{\text{med}\%}\leq k_{W_{\text{med}}C3}\\ \frac{W_{\text{med}\%}-k_{W_{\text{med}}\text{crit}}}{k_{W_{\text{med}}C3}-k_{W_{\text{med}}\text{crit}}} & \text{if}\;k_{W_{\text{med}}C3}<W_{\text{med}\%}<k_{W_{\text{med}}\text{crit}}\\ \;0 & \text{if}\;W_{\text{med}\%}\geq k_{W_{\text{med}}\text{crit}} \end{array}, \end{array}$$ (12)

where *k*_W_med_C1_ is the lowest water content in the feed below which growth ceases due to reduced feed ingestion rate, *k*_W_med_C2_ is the moisture concentration above which the ingestion rate is maximum, *k*_W_med_C3_ is the water concentration above which diffusion of air into substrate and thus the larvae drops, and *k*_W_med_crit_ is the highest water concentration above which oxygen diffusion ceases.

*Air flow rate and O_2_ concentration*. Effect of aeration in the growing environment influences the larval growth through the availability of O_2_ necessary for respiration. A study performed in [19] that compared the larval growth at different air flow rates and thus the available O_2_ concentration used a logistic model to describe the data.

$$\begin{array}{c} r_{A}\left(A_{\text{air}}\right)=k_{r_{\text{max}}A}{\left(1+\exp(\frac{A_{\text{air}}-k_{A_{\text{inf}}}}{k_{A_{\text{trans}}}})\right)}^{-1}, \end{array}$$ (13)

where *k*_r_max_A_ is the maximum rate, $k_{A_{\text{inf}}}$ is the infliction point and $k_{A_{\text{trans}}}$ is the slope. However, in [36] the authors despite highlighting the logistic model, have used a type II function to model the development rate based on the O_2_ concentration. In this work, the influence of O_2_ concentration is considered as a resource necessary for the underlying biological processes and therefore the growth rate in response to the airflow rate can be modeled as

$$\begin{array}{c} r_{A}\left(A_{\text{air}}\right)=k_{r_{\text{max}}A}\frac{A_{\text{air}}}{A_{\text{air}}+k_{A_{\text{half}}}}, \end{array}$$ (14)

where *k*_r_max_A_ is the maximum growth rate under certain high Air flow rate and $k_{A_{\text{half}}}$ is the airflow rate for which the growth rate is reduced by half.

#### Other factors affecting the underlying biological processes

In previous sections, models describing growth, dry mass partitioning, and external factors influencing the growth and development rate were presented. It is also necessary to establish the relation between the development stage of the larvae and how it influences the underlying biological processes such as assimilation, maturity and maintenance. Here, the regulation function describing the relation between the assimilation and maintenance rates due and the current development stage of the larvae is presented.

*Feeding and growth stage*. Larval structural growth is a result of constant assimilation—the main function of the larvae is to accumulate enough assimilates and mass—lasting up-to the 6th instar [26]. According to the results published in [18], the larvae assimilates the feed at highest rates during the 1st to 4th instar. This gradually drops from 4th to 6th instar and assimilation finally ceases before 7th instar. It is also observed in the works of [18], that the mouth parts of the larvae undergo morphological changes suggesting changes in feeding behavior.

With respect to the model considered in this work, this transition into non-feeding stage indicate that the assimilation is highest in the early larval stages, gradually decreases with increase in mass, and finally ceases when the feeding stage is completed. These variations of the assimilation process over the development stages, indicated by *T*_Σ_, can be described as

$$\begin{array}{c} r_{B_{\text{assim}}}\left(T_{\Sigma }\right)=\{\begin{array}{cc} r_{{\text{assim}}_{\text{max}}}\left(B_{\text{dry}}\right) & \text{if}\;T_{\Sigma }<k_{T_{\Sigma }1}\\ r_{{\text{assim}}_{\text{max}}}\left(B_{\text{dry}}\right)(\frac{T_{\Sigma }-k_{T_{\Sigma }2}}{k_{T_{\Sigma }1}-k_{T_{\Sigma }2}}) & \text{if}\;k_{T_{\Sigma }1}\leq T_{\Sigma }<k_{T_{\Sigma }2}\\ 0 & \text{if}\;T_{\Sigma }>k_{T_{\Sigma }2} \end{array}, \end{array}$$ (15)

with $r_{{\text{assim}}_{\text{max}}}\left(B_{\text{dry}}\right)=1-\frac{B_{\text{dry}}}{k_{B_{\text{asy}}}}$, where $k_{B_{\text{asy}}}$ is the maximum asymptotic mass of the larvae, *k*_T_Σ_1_ is the transition point until which the larvae feeds at a maximum rate and *k*_T_Σ_2_ is the point beyond which the feeding comes to a halt. The function $r_{{\text{assim}}_{\text{max}}}$ represents the ingestion/feeding potential of the larvae in relation to it its current size and the maximum size it can reach when infinitely fed. With this Eq (17), a relation between the development sum and the size dependent ingestion is established.

*Maturation stage*. In the final larval instar stage, the accumulated assimilates and reserves (e.g. fats) are further spent in developing the parts necessary to reach the maturity and transform into a pupae. This maturity process was studied in [37] which indicates a drop in the dry mass and, in specific, the crude fats during the transition from prepupae to pupae. However, maturation ceases at the end of this transformation and then the pupal stage begins. This maturity allocation, can be modeled as a rate that allocates the assimilates and reserves to the maturation process. Allocation to maturity can be also seen in the modeling approaches of DEB [38] (see Section 2.4). In this work, such scheduling of assimilates to maturation is done such that the maturity process continues further after the feeding phase and until the larvae turn into pupae. This continued allocation describes the drop in mass and indicates the change in body composition. Therefore, the variation in maturity allocation is described as

$$\begin{array}{c} r_{B_{\text{mat}}}\left(T_{\Sigma }\right)=\{\begin{array}{cc} 1 & \text{if}\;T_{\Sigma }<k_{T_{\Sigma }3}\\ 0 & \text{if}\;T_{\Sigma }\geq k_{T_{\Sigma }3} \end{array}, \end{array}$$ (16)

where *k*_T_Σ_3_ indicates the end of prepupal or beginning of pupal stage. For the model to track pupal development, the maturity allocation shall be replaced with a non zero value since the pupae undergo further metamorphosis consuming reserves.

#### Combining growth, development and external factors

**Fig 2:**
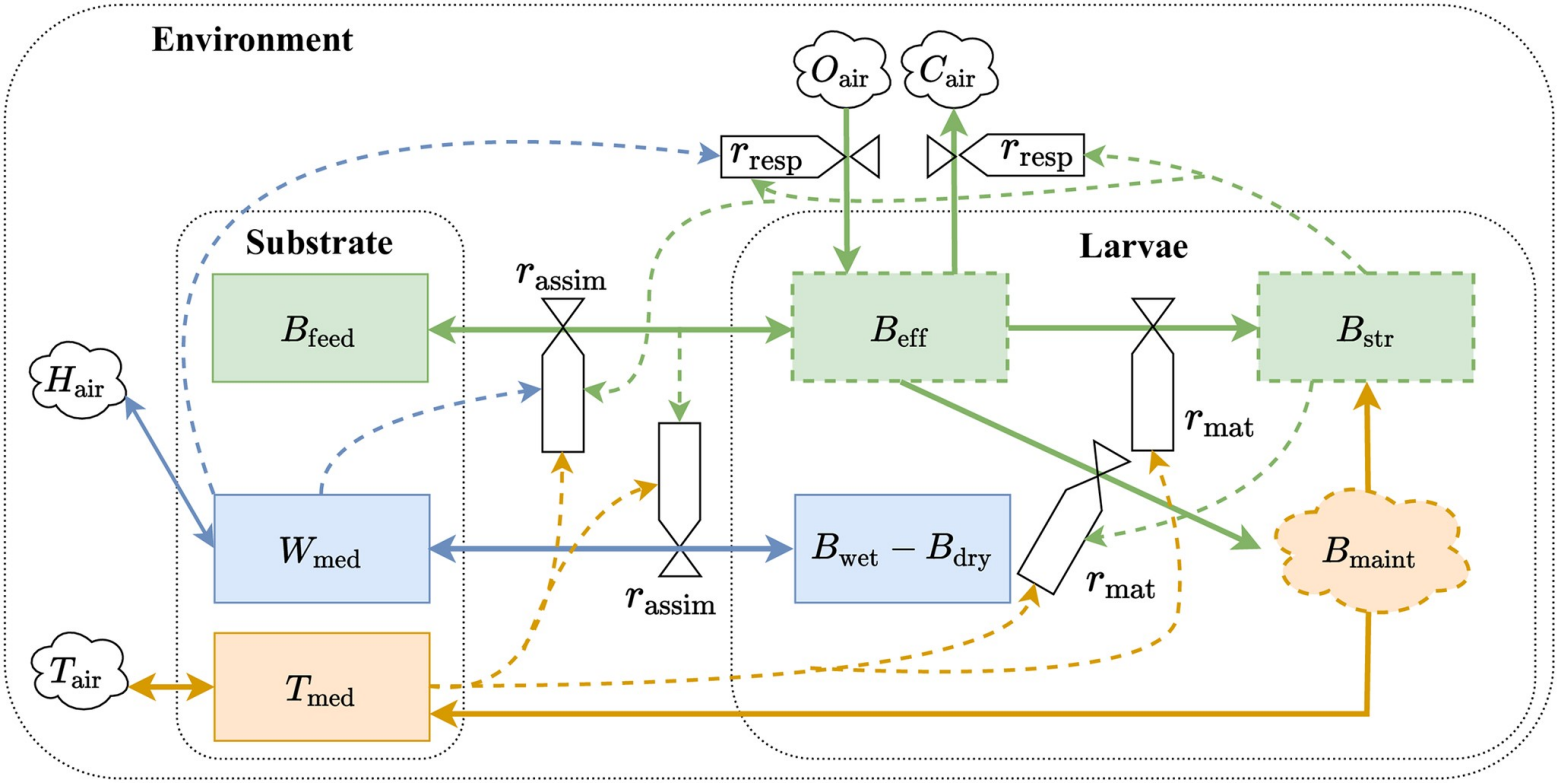
Mass and energy transfer. The flow of mass and energy between the substrate or growing medium, larva body and the environment in response to various states and environment conditions are represented using the valves that regulate this flow. Influence of the states on the rate are indicated using dashed lines. Influence of the states on the rates are not explicitly indicated when the flow takes place between those corresponding states. Valves *r*_assim_, *r*_mat_, and *r*_resp_, represent the assimilation, maturity-maintenance and respiration as a function of various states that influence the flow of biomass and energy.

The factors considered to be affecting the growth of larvae and the movement of mass and energy between larva, growing medium and the environment is summarized in Fig 2 using Forrester diagram. The *B*_str_, representing the structural mass of the larva, has its influences on most of the rate flows as seen in Fig 2.

From Fig 2, it can be seen that the rate or regulatory functions *r*_assim_ and *r*_mat_ acts as valves that regulate the underlying process by controlling the flow of necessary resources. From this context, these regulatory functions can be, in simple way, modelled to vary from to 0–1 such that for best conditions the valves are set to attain the maximum rate possible for the underlying processes. In case of deficiencies in any of the required growing conditions, the rate goes down reducing the rate of the underlying processes. Therefore, the regulatory function for assimilation can be modelled as a product of all rate functions responsible. This can be written as

$$\begin{array}{c} r_{\text{assim}}=r_{B_{\text{assim}}}\left(T_{\Sigma }\right)(\frac{r_{T}\left(T_{\text{med}}\right)}{k_{r_{\text{max}}T}}\;\frac{r_{F_{\text{grw}}}\left(B_{\text{feed}}\right)}{k_{r_{\text{max}}\text{gm}}}\;\frac{r_{W}\left(W_{\text{med}\%}\right)}{k_{r_{\text{max}}W}}\;\frac{r_{A}\left(A_{\text{air}}\right)}{k_{r_{\text{max}}A}}). \end{array}$$ (17)

Similarly the processes underlying maturity-maintenance is affected by temperature for metabolic activities, air concentration for respiration, available reserves indirectly represented by the feed density, and finally the development state of the larvae. Therefore, this can be modelled as

$$\begin{array}{c} r_{\text{mat}}=r_{B_{\text{mat}}}\left(T_{\Sigma }\right)(\frac{r_{T}\left(T_{\text{med}}\right)}{k_{r_{\text{max}}T}}\;\frac{r_{F_{\text{grw}}}\left(B_{\text{feed}}\right)}{k_{r_{\text{max}}\text{gm}}}\;\frac{r_{A}\left(A_{\text{air}}\right)}{k_{r_{\text{max}}A}}). \end{array}$$ (18)

Finally, the regulation function for development rate *r*_dev_ can be modelled similarly to Eq (17), a function of all factors affecting development, as

$$\begin{array}{c} r_{\text{dev}}=(\frac{r_{T}\left(T_{\text{med}}\right)}{k_{r_{\text{max}}T}}\;\frac{r_{F}\left(B_{\text{feed}}\right)}{k_{r_{\text{max}}\text{dm}}}\;\frac{r_{W}\left(W_{\text{med}\%}\right)}{k_{r_{\text{max}}W}}\;\frac{r_{A}\left(A_{\text{air}}\right)}{k_{r_{\text{max}}A}}). \end{array}$$ (19)

Regulation functions Eqs (17) to (19) play a significant role is describing the dynamic interaction between the larvae and its environment.

### Implementation of switching functions

The functions that are modelled in this work as cases or switching functions, Eqs (11), (12), (17) and (18), are realized as logistic functions in the actual implementation of the model in MATLAB to allow for a smooth transitioning between the cases. This is especially done, firstly, to replicate the behavior in biology where the transition is gradual and not a sudden change and, secondly, to allow for the numerical solvers to converge to the solution.

A logistic function modelling the transitions from 0–1 (sigmoid curve) can be written as

$$\begin{array}{c} f_{\text{logi}}\left(x\right)={\left[1+\exp(-k(x-x_{0}))\right]}^{-1}, \end{array}$$ (20)

where *k* defines the slope for the transition between 0 and 1, and *x*_0_ defines the mid point of the transition where the function reaches half of its maximum value. The two parameters for the Eq (20) can be calculated and substituted back to obtain a smooth transitioning version of the function free of kinks. An example for Eq (17) is provided here as

$$r_{B_{\text{assim}}}\left(T_{\Sigma }\right)={\left[1+\exp((\frac{-4}{k_{T_{\Sigma }1}-k_{T_{\Sigma }2}})(T_{\Sigma }-k_{T_{\Sigma }\text{inf}}))\right]}^{-1}$$

with *k*_T_Σ_inf_ = *k*_T_Σ_1_ + 0.5(*k*_T_Σ_2_ − *k*_T_Σ_1_).

### Model validation and parameter estimation

Models presented in this work are mostly nonlinear and therefore, nonlinear least squares data fitting method was used for parameter estimation. This was formulated as an optimization problem with the objective of finding the parameter that minimizes the sum of square of errors as

$$\begin{array}{c} \min_{p}\;\sum_{i}{\left(f\left(p,X_{i}\right)-Y_{i}\right)}^{2}, \end{array}$$

where **p** represents the parameters to be estimated, *f*(**p**, **X**) represents the model, **Y** is the measured data and *i* represents the measurement samples. This parameter estimation problem was implemented in MATLAB using the *lsqcurvefit* function in a multi-search framework to explore possible solutions within the specified boundary values of parameters. Simulation of dynamic model was performed using the *ode*45 solver in MATLAB to solve the differential equations.

The data sets obtained from different literature sources, as presented in Table 2, were used in this work for the purpose of parameter estimation and validation. Steps followed in preparing the data, estimating the parameters of the static models Eqs (6) to (10), (13) and (14), and using them in the final models are described as below:

1. Identify the datasets for the constant and variable factors. For example, for data set T1, temperature is variable but feed type and other factors are constant. Similarly, T2-T4 have variable temperatures but other factors are constant within the data sets. Therefore, T1-T4 from common data sets serving the same purpose (Temperature dependency).
2. Group together the data sets serving common purpose and normalize the measurement values. This converts the data sets to a common scale and remove any dependency due to other variables between the data sets. For example, feed type used in the data set T1 is different from T2, T3 and T4. Therefore, dividing the development and growth rates by the observed maximum value will transform the measurement of all data sets (T1-T4) to a common scale (0-1).
3. Perform the parameter estimation using the individual normalized data sets and also the normalized data sets grouped together.
4. Parameters estimated using the grouped data sets, later labelled as average data, are used as final parameters for the model.
5. Finally, to transform the result (growth and development rates) back to the actual scale (s^-1^ or g s^-1^), the results are multiplied by the maximum rate observed in the data sets. These maximum rates observed, corresponds to the parameter *k*_r_max_XYZ_ where _XYZ_ represents individual static models.

**Table 2:** Data sets and their source used for model validation and parameter estimation.

Dataset ID	Source	Description	Application
T1, T2	Fig 3 of [10]	Development time of BSF on different diets at different temperatures	Validation and parameter estimation for Eqs (6) and (7)
T3, T4	Table 1 of [11]		
F1	Table 2, 3 of [13]	Development time and dry weight respectively of BSF larvae under different feeding rates	Validation and parameter estimation for Eqs (8) and (9)
F2-F4	Table 2, Fig 1 of [39]	Development time and dry weight respectively of BSF larvae under different feed and feeding rates	
M1	Fig 4 of [19]	Larvae growth/dry weight change under different substrate moisture content	Validation and parameter estimation for Eqs (10) and (12)
M2	This work		
M3	Table 2 [35]		
A1	Fig 2 of [19]	Larvae growth/dry weight change under different aeration rate	Validation and parameter estimation for Eqs (13) and (14)
G1	Fig 1 of [37]	Larvae growth/dry weight change over the developmental phases	Validation and parameter estimation for Eqs (4), (17) and (18)
D1, D5	Fig 2 of [13]	Larvae growth/dry weight change over the developmental phases under different feeding rates	
D2-D4	Fig 2 of [13]	Larvae growth/dry weight change over the developmental phases under different feeding rates	Validation of Eq (4)

Parameters corresponding to the dynamic model Eqs (4) and (5) are estimated using measurement data that contains larvae dry mass change over time and the corresponding growing conditions maintained during the entire time. Some of the parameters obtained from the static models were re-estimated in order to accommodate the dynamics and any changing growing conditions. Once re-estimated for one data set, the parameters should be valid for all the data sets from the data sets grouped together. This step calibrates the dynamic model for the new data sets. For example, in this work, parameter values for *k*_B_half_gm_ and *k*_B_half_dm_ were re-estimated to calibrate the dynamic model for the new datasets D1-D5, since the feed type changed. However, this re-estimation was only performed using a subset of the available data sets (D1 and D5). The calibrated models, using the new estimated parameter value, shall be now valid for all data sets D1-D5. Results obtained using the above described procedure and the resulting quality of fit for each data set is presented in the following section.

## Results

In this section, firstly, the performance of the individual rate functions Eqs (6) to (10), (13) and (14) describing the influence of external factors on growth and development are presented. Secondly, performance of the combined dynamic model representing the growth Eq (4) and development Eq (5) are presented, highlighting the validity of the rate functions Eqs (17) and (18) for assimilation and maturation respectively. Finally, the dynamic growth and development model are validated using additional datasets.

### Temperature influence

**Fig 3:**
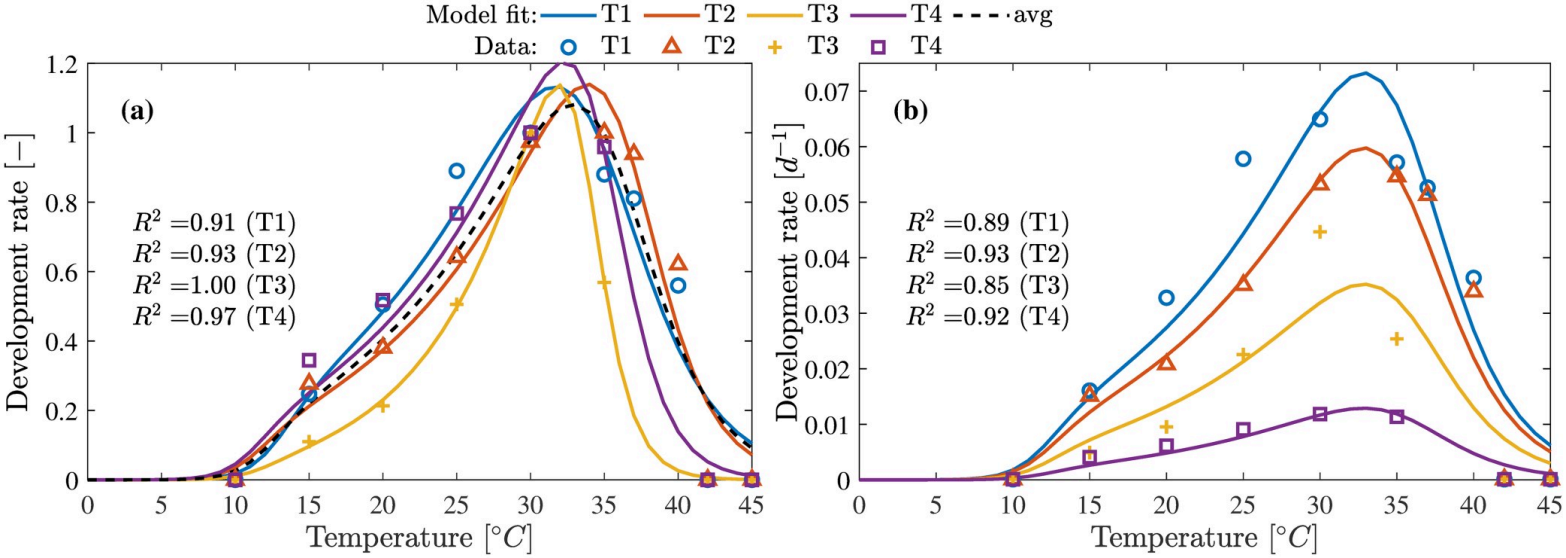
Temperature influence on development rate using Arrhenius model. **(a)** Parameters estimation using Arrhenius model (6) and normalized data. **(b)** Development rate estimation using the parameters estimated for data set obtained by averaging all (T1-T4) data sets. Model fit represented as avg shows the performance of the final model.

**Fig 4:**
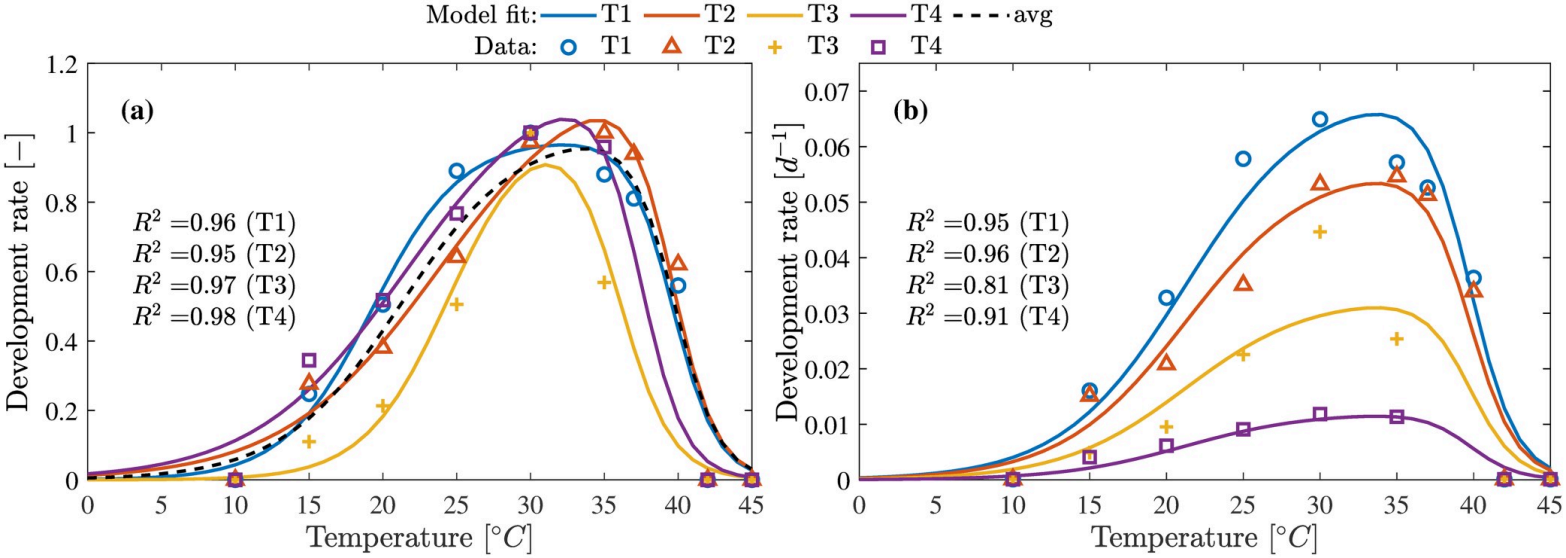
Temperature influence on development rate using modified Logan-10 model. **(a)** Parameters estimation using modified Logan-10 model (7) and normalized data. **(b)** Development rate estimation using the parameters estimated for data set obtained by averaging all (T1-T4) data sets. Model fit represented as avg shows the performance of the final model.

A total of four data sets (T1-T4) representing the influence of temperature on larvae development was obtained from [10, 11]. These four data sets represent the temperature dependency under four different feed types and was used to obtain the parameters for models Eqs (6) and (7). Fig 3 shows the results of the parameter estimation using the Arrhenius model (6) and Fig 4 for the modified Logan-10 model Eq (7).

Both models perform well in describing the data with good quality of fit (*R*^2^ > 0.91). The model parameter obtained from the average data set was used to explain the data sets T1-T4 as shown in Figs 3(b) and 4(b). The results of the model with the estimated parameters from the average data, performed well in explaining all data sets with the only exception for T3 where both models could not explain the peak at 30°C. Modified Logan-10 model has overall better quality of fit for both normalized and actual data sets. For temperatures below 15°C, Eq 6 provides a better fit at the expense of one additional parameter.

### Feed density

**Fig 5:**
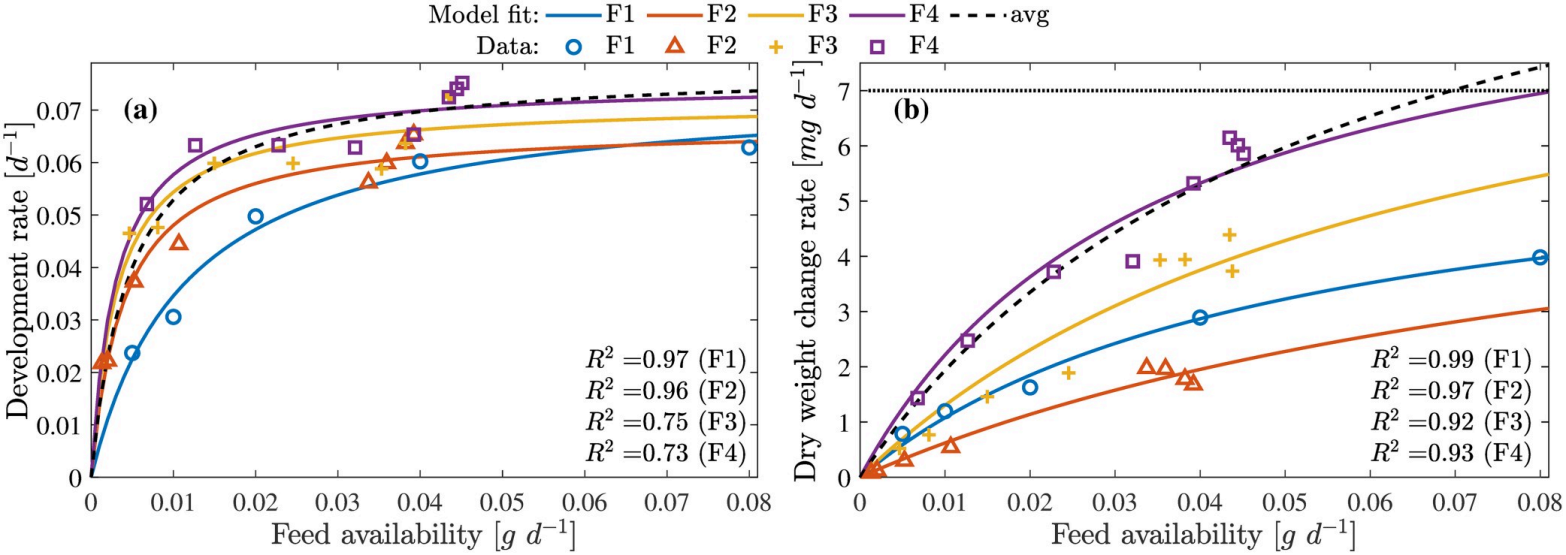
Feed availability on development and growth. **(a)** Larvae development rates at varying feed availability. **(b)** Larvae growth rates at varying feed availability. Model fit avg represents the results of the average model scaled to the maximum observed development rate from the 4 data sets.

**Fig 6:**
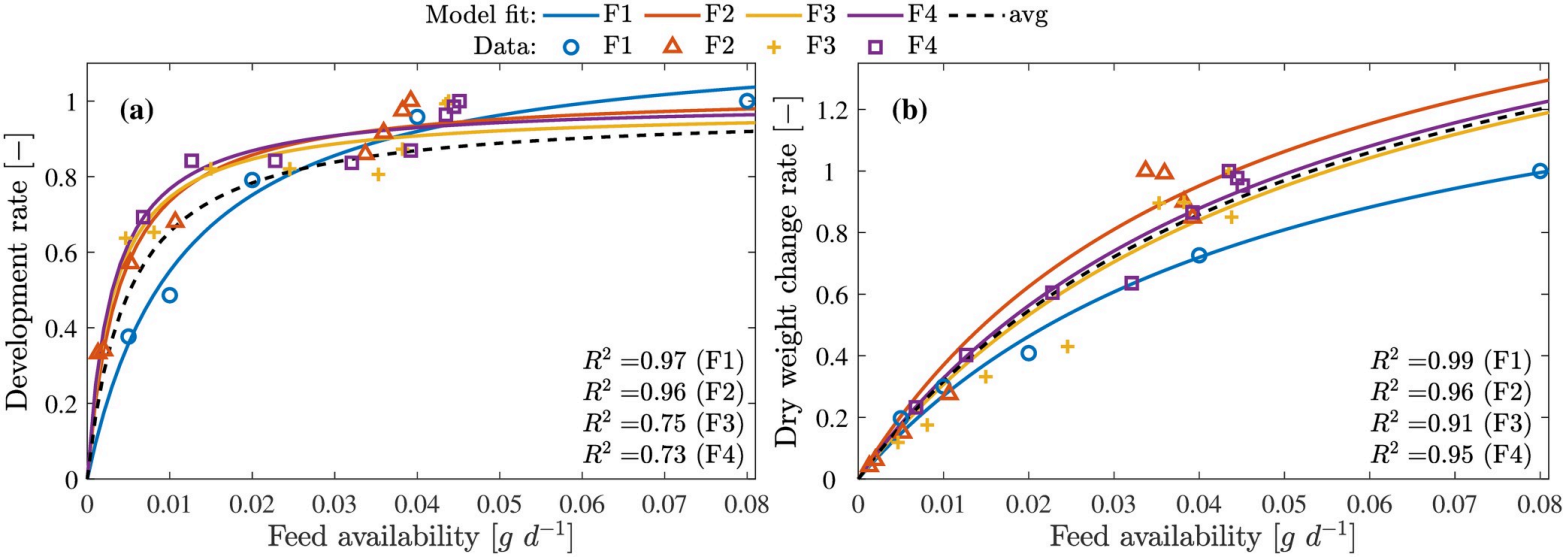
Feed availability on development and growth (Normalized). **(a)** Larvae development rates at varying feed availability. **(b)** Larvae growth rates at varying feed availability. Mode fit avg represents the results of the average model for the combined data sets.

Results published in [13] was used to obtain data set F1 and [39] for data sets (F2-F4) representing the development and growth rate under different feeding densities and feed types. The feed density defined in these works use gram dry mass of feed available/provided per larvae per day (g d^-1^ per larva) during the feeding periods. These data sets were used to obtain the development rates and growth rates using the model Eqs (8) and (9) as shown in Figs 5 and 6.

The models describe accurately (*R*^2^ = 0.97) for the data set F1 due to the availability of measurement for uniformly distributed feed densities. In case of F2-F4, the data set also includes both batch fed and continuous fed experiment measurements resulting in scattered measurements and thus lower quality of fit. Growth rate model, on the contrary, performs better in describing all data sets F1-F4 with *R*^2^ > 0.92. The parameters for these models are obtained by combining the normalized data sets F1-F4. The resulting model from this combined data is shown with the dashed line (indicated as avg) as in Fig 6. From these results it could be concluded that these models can be used to compute the growth and development rates under different feeding rates.

### Moisture effect on growth

**Fig 7:**
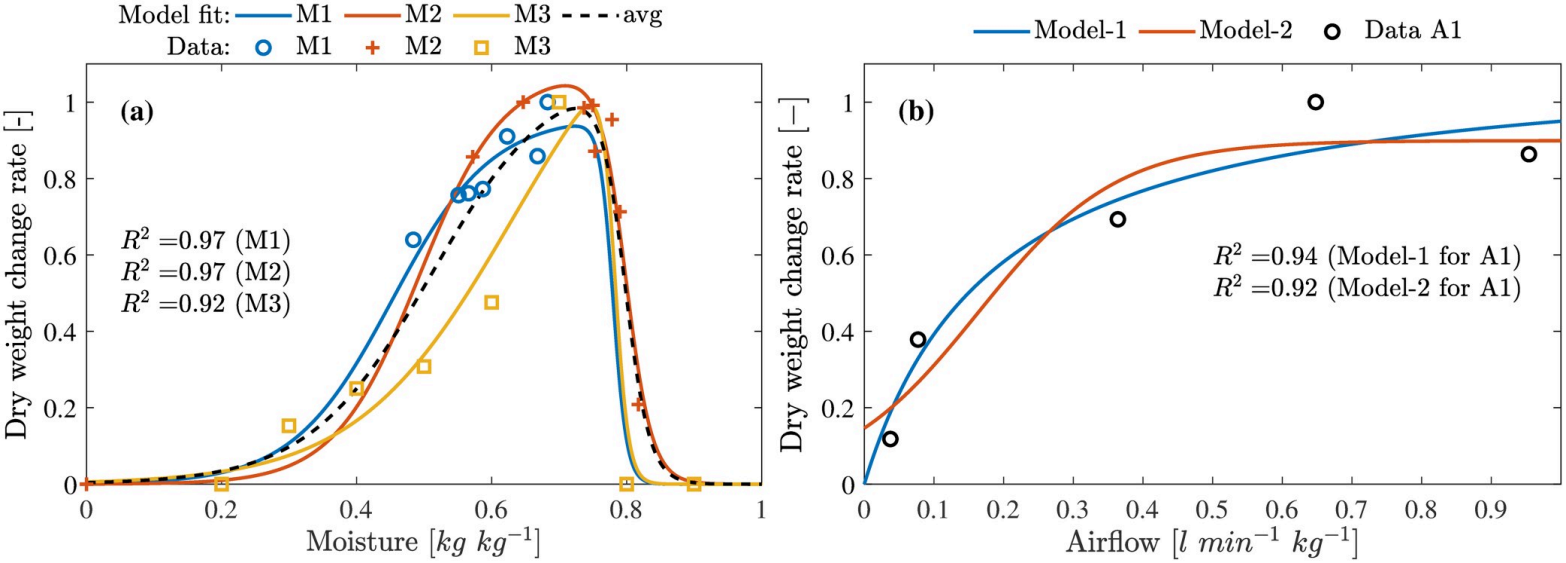
Moisture and airflow on growth rate. **(a)** Effect of substrate/feed moisture on the growth rate. **(b)** Effect of airflow rate on the growth rate in closed production. Model-1 represents the Monod model Eq (14) and Model-2 represents the logistic model Eq (13).

To evaluate the model presented in Eq (10), for influence of moisture on the growth, a total of three data sets M1-M3 were obtained. M1 and M3 are results published in [19] and [35] respectively. Data sets M1 and M2 does not contain measurements for the entire moisture concentration range but M3 provides data for the range from 20% to 90% as seen in Fig 7(a). The proposed model is capable of describing the growth for the considered data sets and also the observations from the moisture experiment coincides with [35] for the higher moisture concentration. On the contrary, higher development rate was observed for moisture at 80% in [40]. Further investigation with complementary data sets may be necessary to identify the boundaries for higher moisture concentrations.

### Airflow rate

Only one literature was found that included the effect of airflow on the growth of the larvae in [19]. In that work, closed 750 mL bioreactors with different aeration rates were used to study the larval growth rates. As expected, in closed environment, growth was slow at lower aeration rates and increased gradually with increasing aeration rates and finally saturates. Model Eqs (14) and (13) were evaluated and the results are presented in Fig 7(b). From these results, one can see that both models can describe the growth under various aeration rates. However, Eq (14) provides better results for the available data sets. Further studies might be necessary to obtain the growth response to different flow rates under varying moisture concentrations to identify correlation between them.

### Larvae growth and development

**Fig 8:**
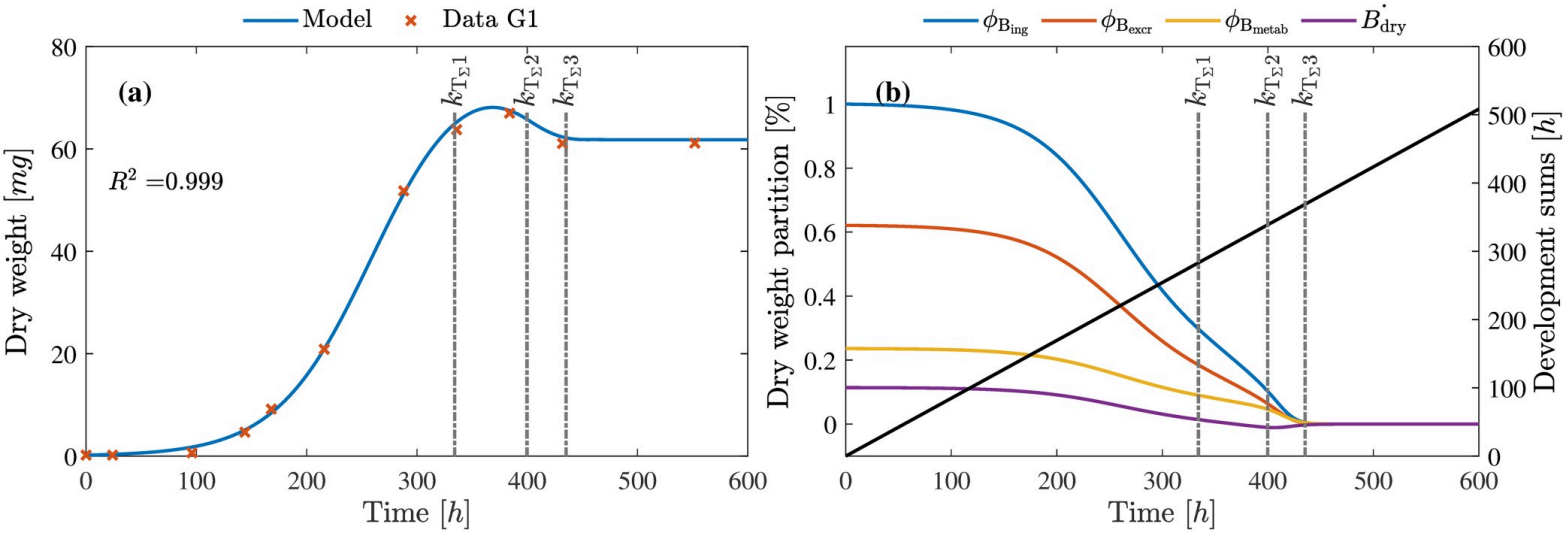
Larvae growth, development and biomass partitioning. **(a)** Larvae dry mass evolution over time **(b)** Partitioning of assimilates and dry mass over development phases. The development sum where the assimilation and maturity transition, are indicated by the horizontal markers labelled *k*_T_Σ_1_, *k*_T_Σ_2_, and *k*_T_Σ_3_.

The larvae growth model presented in Eq (4), describing the evolution of dry mass of the larvae, and the development model presented in Eq (5) was validated based on the dry weight measurements presented in literature [13] and [37]. Dry mass of the *Hermetia illucens* from eggs to adult, presented in [37], was used to obtain estimates of the parameters marking the important stages *k*_T_Σ_1_, *k*_T_Σ_1_, and *k*_T_Σ_3_. Based on the biomass conversion efficiency for chicken feed provided in [8, 13], parameters for dry mass distribution to metabolism, excretion and growth are also estimated to 0.24%, 0.62%, and 0.11% respectively. The performance of the model based on the estimated parameters is presented in Fig 8.

Initially, the change of larval dry mass from the 1st larval instar to the 5th instar is regulated by the asymptotic size of the larva (in dry mass) as seen between 0-320 h marked by *k*_T_Σ_1_. As the larvae approaches its last instar, ceasing of the ingestion process marked by the morphological changes such as modification of mouth parts and darkening of the skin are identified by the *k*_T_Σ_2_ and *k*_T_Σ_3_. The final transition from prepupae to pupae is marked at the end of *k*_T_Σ_3_, indicating the end of larval growth and start of pupal stage.

**Fig 9:**
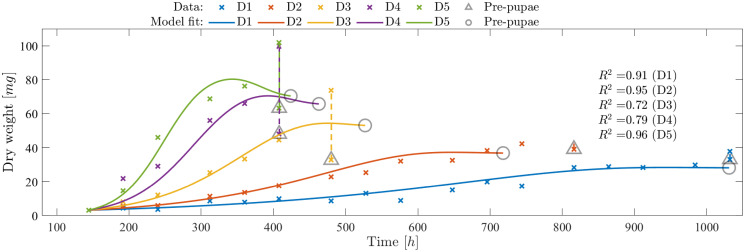
Validation of larvae growth and development model. Models are validated based on the data sets D1-D5 as published in [13]. The vertical line indicates the time point where about 50% of the larvae are transformed into prepupae. The circle on the corresponding model fit indicate the *k*_T_Σ_3_ time point when development of larvae are completed.

Furthermore to validate the model for different data sets, data set from [13] was used. Using the data sets D1 and D5, model parameters were further adjusted for the new setup and using these new parameters the performance of the model was validated for all data sets D1-D5. The results as seen in Fig 9, highlights the performance of the model by providing the dry weight evolution as well as the indication of the different Larval development stages.

As seen from Fig 9, the *R*^2^ for the data sets D1,D2 and D5 are > 0.91 but comparatively lower for D3 and D4. This is purely due to the variance in the final recorded weight for D3 and D4. To support this inference, we can also observe that for D3, D4 and D5 there were larvae with higher mass on the same day when 50% prepupae were observed. The Eq (4) also models higher assimilates allocation to maturity for higher feed density, indicating that more reserves are available in larvae growing in higher feed density. This can be seen in the drop in dry mass when larvae transforms to prepupae. This drop, as explained by the model and also as observed from the data is highest for D5 and lowest for D1.

### Summary

From these observation one can conclude that the dynamic model developed in this work serves the two main intended goals: (1) model the larval growth through change of dry mass *B*_dry_ over its development stages under different growing conditions; and (2) model the transition of larval development through the development sum *T*_Σ_ under different growing conditions. These results were achieved using simple model structures which plausibly and consistently explained various data from the literature. Finally, the model parameters estimated in this work are summarized in Table 3. Data set used for parameter identification of the moisture influence and the matlab implementaion of the model are included as supplementary information (see S1 Dataset and S1 File).

**Table 3:** Estimated model parameter values.

Parameter	Est. value	Parameter	Est. value	Parameter	Est. value
$k_{r_{\text{ref}}T}$ (6)	0.7195 *	$k_{T_{A}}$ (6)	8450 K	$k_{T_{\text{AL}}}$ (6)	60000 K
$k_{T_{\text{AH}}}$ (6)	40667.275 K	$k_{T_{\text{ref}}}$ (6)	298.92 K	$k_{T_{L}}$ (6)	285 K
$k_{T_{H}}$ (6)	308.96 K	$k_{r_{\text{max}}T}$ (7)	1.0*	$k_{r_{\text{base}}T}$ (7)	0.215*
$k_{\rho }T$ (7)	0.2487°C^-1^ d^-1^	$k_{T_{\text{max}}}$ (7)	39.769°C	$k_{\Delta }T$ (7)	3.0°C
$k_{T_{\text{base}}}$ (7)	15.95°C	$k_{B_{\text{half}}\text{dm}}$ (8)	0.0049 g d^-1^	$k_{r_{\text{max}}\text{dm}}$ (8)	0.9758*
$k_{B_{\text{half}}\text{gm}}$ (9)	0.00532 g d^-1^	$k_{r_{\text{max}}\text{gm}}$ (9)	2*	$k_{W_{\text{med}}C1}$ (10)	0.329 kg kg^-1^
$k_{W_{\text{med}}C2}$ (10)	0.69 kg kg^-1^	$k_{W_{\text{med}}C3}$ (10)	0.76 kg kg^-1^	$k_{W_{\text{med}}\text{crit}}$ (10)	0.833 kg kg^-1^
$k_{r_{\text{max}}A}$ (14)	1.128*	$k_{A_{\text{half}}}$ (14)	0.1877 l min^-1^ kg^-1^	$k_{B_{\text{half}}\text{dm}}$ (8)	0.0137 g d^-1^+^^
$k_{r_{\text{max}}\text{dm}}$ (8)	1* ^+^	$k_{B_{\text{half}}\text{gm}}$ (9)	0.0717 g d^-1^+^^	$k_{r_{\text{max}}\text{gm}}$ (9)	1* ^+^
$k_{\alpha_{\text{excr}}}$ (2)	0.5762	$k_{\alpha_{\text{assim}}}$ (2)	0.2135	$\epsilon_{\text{inges}}$ (2)	0.79
$k_{\text{inges}}$ (4)	1.61 × 10^-4^ g g^-1^ s^-1^	$k_{\text{maint}}$ (4)	5.6779 × 10^-6^ g g^-1^ s^-1^	$k_{T_{\Sigma }1}$ (15)	234.35 h
$k_{T_{\Sigma }2}$ (15)	265.5 h	$k_{T_{\Sigma }3}$ (15)	297.5 h	$k_{B_{\text{asy}}}$ (15)	0.115 g

* values are normalized.

^+^ parameters re-estimated for data set D1 and D5.

## Discussion

Growth and development of *H*. *illucens* larvae depend on several internal and external factors. Quantitative study of these factors and their influence is necessary for a better understanding of the growth dynamics and eventually its application for solving some of the real-world problems (e.g. rearing for feed, waste processing etc.). There have been several studies that explore the application of *H*. *illucens* larvae to recycle food waste [6–8, 13, 41–47] and also in production of alternative animal feed [1, 2, 4, 48–50]. Such exploration for suitable application have also led to studies to understand the various factors affecting the growth and identifying the suitable conditions for larvae rearing [9–16, 20, 39–42, 51–57]. However, quantification of these interaction between the larvae and their growing conditions using models can only be seen in few literatures with studies limited to temperature and diet quality [10, 13, 20, 42].

This study has extended the scope of mathematical models to not only temperature but also other factors such as moisture in feed, feed density and air flow rates. Using Arrhenius and modified Logan10 model, the influence of temperature on the metabolic rates driving the growth process over a wide temperature ranges were shown. It could be seen from Figs 3 and 4 that the larvae have highest growth rates between 30-35°C. Despite showing the variation of the growth rates over the temperature ranges, the models also captures the death of the larvae under extreme temperatures (12°C > *T*_med_ > 40°C).

It is shown in [13, 39, 40] that the larvae grow faster with increase in feed availability and moisture. Growth rate, however, saturated with higher feed availability but significantly dropped at higher moisture concentration indicating larvae death. This interaction indicates that larvae ingest moist feed faster and the ingestion rate is limited by the size of the larvae mouth parts. Therefore, growth rate saturated despite excess feed being available. This is explained well by the model Eq (9) as shown in Fig 5 indicating that the feeding rate saturates despite increase in feed availability. The dependency of feed moisture modeled by the Eq (11), describes increasing ingestion rate with increasing moisture (0.25 g g^-1^ > *W*_med%_ > 0.75 g g^-1^). This relationship between the larvae, feed availability and feed moisture is explained by the model presented in this work as seen in Fig 7(a).

Similar to any other resources consumed by the larvae for its growth, O_2_ concentration in the growing medium also plays as significant role as show in [19]. Since larvae grows in moist feed, factors such as depth and water concentration of the feed plays an important role in gas exchange. Similar to feed availability, growth rate increases with increase in air-flow rate but saturates at higher flow rate despite an increase in the flow rate as show in Fig 7(b). Also, as shown in [35], increasing the water concentration in feed could causes the larvae to drown and result in death due to decreased gas exchange. These dependencies between, water concentration, gas exchange, respiration and air-flow rate are captured in Eqs (12) and (14). From the results presented in Fig 7(a), it can be seen that the growth rate drops when moisture is > 0.75 g g^-1^ indicating mortality at higher moisture concentrations.

Finally, the main focus of this work, to track the growth and development of the larvae over its lifetime (from neonate to pupae), dynamic model Eqs (4) and (5) are presented. The static model presented in [20] used a black-box modelling approach and only showed the larvae growth based on a single factor (i.e. diet quality). However, as shown in this work, it is important to consider other factors affecting the growth and development. Also, it is important to consider a dynamic modelling approach since it allows for accommodating the changes that take place within the larvae and their growing environment over the entire growth phase. These dynamics, as described using the regulation functions Eqs (17) to (19), explain the variation in the internal metabolic processes in response to the growing environment and the development stages. Results presented in Fig 8 show that the model proposed in this work describes the dynamic changes in larval dry mass over its growing phases, highlighting the conversion of feed into biomass. In addition, as shown in Fig 9, the model is also capable of accurately describing the growth, development time, and transition phases under different growing conditions aligning with the results presented in [13].

The models presented in this work have a broad application potential. All static models presented in this work can be used to compute certain static information that can be useful in planning the rearing process and designing the rearing environment. For example, temperature and air-flow rate dependency model, Eqs (6), (7) and (14), could be very useful in determining the heating, cooling, and air-flow requirements. Such information could be used to deduce requirements, in mass production context, for designing suitable reactors. Similarly, feed density and moisture models, Eqs (9) and (10), could be useful in preparing the feeding regimes and feed recipes for either better growth rate and reduced larval mortality or for better waste processing. The dynamic model, Eqs (4) and (5), serve as tools to compute dynamic information pertaining to the larvae growth and development. With these models, for any given sequence of growing conditions (changing over time) and initial larvae weight, it is possible to estimate/simulate the evolution of the larval dry mass and the resources consumed during the growth. Such simulations provide better understanding of the underlying process dynamics that are useful in designing algorithms that can control the reactors. Such models could also be used together with the dynamic models of the reactors to perform static and dynamic optimization of the growing process. Such optimization could be specifically designed for improved energy and resource efficiencies, in mass production context, as shown in [58].

## Conclusion

Based on comprehensive data sets aggregated from literature, various factors that affect the growth and development of larvae were analysed. Models were developed based on literature and based on the analysis of data to accurately and plausibly describe the influence of different environmental conditions such as temperature, feed density, feed moisture, and airflow rate on growth and development rates of BSF larvae. Building on the principles of mass balance, von Bertalanffy and DEB models, a novel dynamic model describing the growth and development of *Hermetia illucens* larvae was developed. Concept of development sum was proposed to establish a relationship between growth and development. The comprehensive dynamic model was obtained consisting of two differential equations, larval dry mass *B*_dry_ and development sum *T*_Σ_, and combining the different rate equations. Model parameters were estimated for all the proposed models based on extensive data sets from different literature and the models were validated with *R*^2^ > 0.90 with only few exceptions. The resulting dynamic model describing the growth and development of the *Hermetia illucens* larvae was validated, using parameters obtained from only a subset of data, on all available data sets. The dynamic model, proposed and validated in this work, consistently explained: the change of larval dry mass over time; transition of development phase and its effect on growth; and influence of external factors on the larval growth and development. Performance of the model could be further improved with newer and larger data sets that could reveal other mechanisms or biological processes not explored in this work. Extension of this work with considerations for energy and resource efficient production of *Hermetia illucens* larvae in large scale production environments will be the future goal.

## Supporting information

S1 Dataset Experiment data. This file contains the data for the moisture dependency experiment executed in this work.
(XLSX)

S1 File MATLAB files for model. The package contains MATLAB implementation of the dynamic model presented in this work.
(ZIP)
